# Evaluation of rs781673405, rs1244378045, rs767450259, rs750556128, rs369143448, rs143353036, and rs759369504 mutations in terms of polymorphism in diabetic obese and non-diabetic obese individuals

**DOI:** 10.1007/s12020-025-04184-0

**Published:** 2025-02-22

**Authors:** Saadet Busra Aksoyer Sezgin, Sermin Durak, Faruk Celik, Varol Guler, Aysegul Sarikaya, Umit Zeybek

**Affiliations:** 1 Faculty of Medicine, Department of Medical Biology and Genetics, Istanbul Yeni Yuzyil University, 34010 Istanbul, Turkey; 2 Faculty of Medicine, Department of Medical Microbiology, Istanbul University Cerrahpasa, 34320 Istanbul, Turkey; 3 Department of Molecular Medicine, Aziz Sancar Institute of Experimental Medicine, Istanbul University, 34093 Istanbul, Turkey

**Keywords:** Obesity, Diabetes, Polymorphism, TRAIL

## Abstract

**Background:**

Obesity is among the important healthcare issues in which there is an abnormal increase in body fat because energy intake is higher than energy expenditure. The Tumor Necrosis Factor (TNF)-alpha overexpression in adipose tissue plays important roles in mediating obesity and insulin resistance. “TNF-related apoptosis-inducing Ligand (TRAIL(TNSF10))”, which is a member of the TNF family, is expressed as a Type-II Transmembrane Protein with an effect on the development of obesity and diabetes.

**Methods:**

The rs781673405, rs1244378045, rs767450259, rs750556128, rs369143448, rs143353036, and rs759369504 polymorphisms of TRAIL, which were determined to play protective roles against diabetes, were evaluated with the RT-qPZR Method in the present study.

**Results:**

It was found that the genotype distribution of TRAIL rs767450259 Polymorphism was significant and the T-Allele had protective effects against diabetic obesity. It was also found that the G-Allele of the rs369143448 Polymorphism had protective roles against diabetic obesity. It was shown that carrying the A-Allele in the rs750556128 Polymorphism might increase the risk of obesity in diabetic patients by 1.3-fold.

**Conclusions:**

The present study makes a significant contribution to the literature data because it is the first to investigate these polymorphisms.

## Introduction

Obesity is one of the significant health issues characterized by an abnormal increase in body fat due to energy intake being higher than energy expenditure. In addition to increasing the risk of mortality, this condition leads to various diseases such as diabetes, cardiovascular diseases, kidney diseases, and cancer, as well as psychosocial disorders in individuals [1]. Adipocyte hypertrophy, which develops as a result of excessive food intake, causes adipose tissue dysfunction, which in turn mediates the development of diabetes [2]. Diabetes is a metabolic disease caused by a deficiency in insulin levels released from beta cells in the pancreatic islets of Langerhans or changes in the mechanism of insulin action [3, 4]. The insufficiency in insulin secretion in peripheral tissues mediates the development of insulin resistance by reducing glucose uptake in muscle and fat tissue. In the pathogenesis of diabetes, the primary issue is insulin resistance, alongside the increase in body mass index due to higher food intake, an inactive lifestyle, and environmental and genetic factors [5].

Tumor necrosis factor (TNF)-alpha plays a significant role in mediating obesity and insulin resistance through its excessive expression in adipose tissue [6]. Members of the TNF family hold a crucial place in the pathogenesis of obesity-related diseases, with one such member, the “TNF-related apoptosis-inducing ligand (TRAIL (TNSF10)),” being expressed as a Type-II transmembrane protein. Additionally, TRAIL consists of four transmembrane receptors—TRAIL-R1, TRAIL-R2, TRAIL-R3, and TRAIL-R4—and one soluble receptor, osteoprotegerin [7]. Among these receptors, TRAIL-R1 and TRAIL-R2 lead to the downregulation of peroxisome proliferator-activated receptor-γ by inactivating it through caspase activation. This results in the decreased expression of genes responsible for regulating lipogenesis, ultimately leading to the development of insulin resistance [8].

In addition to its physiological and pathological roles, there is growing evidence supporting TRAIL’s effectiveness in the development of obesity and diabetes, as well as its protective role against diabetes [9–11]. Aksoyer Sezgin et al. show us TRAIL gene expression was compared between the groups which is fold change explained this protective role for example diabetic obese: 0.25; non-diabetic obese:1.68; control 10.95. These results answered the gene expression level increased 10-fold in the control group (*p* < 0.001). Studies modeling diabetes have indicated that TRAIL deficiency and blockade increase the incidence of Type-I diabetes, lead to beta cell dysfunction, affect glucose tolerance, and cause hyperinsulinemia [10, 12].

A study on the rs1131580 single nucleotide polymorphism (SNP) of the TRAIL gene reported that the CC genotype could be associated with diabetes and may also serve as a candidate for prognostic evaluation [13]. However, since studies examining TRAIL gene polymorphisms in relation to diabetes and obesity are quite limited, further research is needed in this area. Based on our literature review, no studies have been conducted on any disease groups concerning the rs781673405, rs1244378045, rs767450259, rs750556128, rs369143448, rs143353036, and rs759369504 single nucleotide polymorphisms of the TRAIL gene. The evaluations of polymorphisms observed in these gene regions have been examined for the first time in this research.

## Material and methods

### Samples used in the study

Our study included 80 diabetic obese people, 80 non-diabetic obese, and 80 healthy people for a control group. They were chosen from the Department of Internal Medicine, Division of Endocrinology and Metabolism, Istanbul University Faculty of Medicine Hospital. The research was carried out at Istanbul Yeni Yuzyil University, Faculty of Medicine, Department of Medical Biology and Genetics. The first group included those who were diagnosed with diabetes and met the obesity criteria. The second group was made up of diabetics who did not have obesity, but did not have any chronic and cardiovascular diseases other than these. The third group consisted of healthy individuals who didn’t have obesity, diabetes, or any chronic disease, not receive any medication under chemotherapy or radiotherapy and not pregnant. The required ethics approval for the study was obtained from the Ethics Committee for Research in Science, Social Sciences, and Non-Interventional Health Sciences at Istanbul Yeni Yüzyıl University (2023/06-1077, 06.06.2023).

### Nucleic acid isolation

2 × 5 ml peripheral blood samples were taken from each individual in the groups. Nucleic acid extractions were performed using the Robotic Device (RINATM M14) (Bioeksen, Turkey). The working principle of this device is based on the isolation of genetic materials by holding them on the surface of magnetic beads, physically and chemically breaking them down, and then extracting them from the cell through enzymatic applications combined with thermal processing, followed by washing. This stage was carried out in accordance with information obtained from literature data [14].

### Analysis of biochemical parameters

Blood samples obtained from the study groups were centrifuged at + 4 °C and 4500 g for 15 min. The analysis of glucose, urea, creatinin, LDH, ALT, AST, total cholesterol, triglyceride, HDL, LDL and HbA1c from the serum samples was performed by using AU5800 Series Clinical Chemistry Analyzers (Beckman Coulter Inc., Brea, CA, USA).

### Primer design, RT-qPCR mehod and RT-qPCR setup

The nucleotide sequences of the primers designed to amplify the region where the polymorphism is observed in each gene region are as follows.

**rs781673405**

OLIGO startlentmgc%any_th3’_thhairpinseq

LEFT PRIMER 37 16 59.52 68.75 14.08 9.63 61.12 TTGGCTGCACCGGCTG / TTGGCTGCACCGGCTC

RIGHT PRIMER (CR) 149 20 58.83 55.00 0.00 0.00 0.00 CAACCGAGATGCTGGACATG

INTERNAL OLIGO 60 20 60.03 55.00 0.00 0.00 0.00 TCTCTTCGACATTCGGCTCC

SEQUENCE SIZE: 268

INCLUDED REGION SIZE: 268

**rs1244378045**

OLIGO startlentmgc%any_th3’_thhairpinseq

LEFT PRIMER 4 26 59.57 38.46 16.65 0.00 36.53 GTTACCTGAGAGGTTCTCTTAATCAT / GTTACCTGAGAGGTTCTCTTAATCAA

RIGHT PRIMER (CR) 111 20 57.08 50.00 0.00 0.00 0.00 TCAGCTAGATCAGCGATAGG

INTERNAL OLIGO 41 26 57.11 26.92 0.00 0.00 0.00 ATAGAATATTGCTTCTAAATTACCCT

SEQUENCE SIZE: 172

INCLUDED REGION SIZE: 172

**rs767450259**

OLIGO startlentmgc%any_th3’_thhairpinseq

LEFT PRIMER W/M 6 25 59.45 40.00 16.65 16.58 41.78 TAGAATTGGAATCCTCAGACGTTCT/ TAGAATTGGAATCCTCAGACGTTCA

RIGHT PRIMER (CR) 121 20 57.08 50.00 0.00 0.00 0.00 TCAGCTTGATGACCCATAGG

INTERNAL OLIGO 51 26 57.11 26.92 0.00 0.00 0.00 ATTGATTAAAGCTTCTTAATTTGCCT

SEQUENCE SIZE: 144

INCLUDED REGION SIZE: 144

**rs750556128**

OLIGO startlentmgc%any_th3’_thhairpinseq

LEFT PRIMER 12 26 59.51 38.46 0.00 0.00 41.78 CTTCAACAGTAGAAATCCTTTCCTCA / CTTCAACAGTAGAAATCCTTTCCTCG

RIGHT PRIMER (CR) 234 20 59.15 55.00 0.00 0.00 0.00 ATTAATCCGAGCTCGCACCC

INTERNAL OLIGO 194 20 59.43 55.00 0.00 0.00 0.00 GCATAGGGTCTGTAAGGGGT

SEQUENCE SIZE: 236

INCLUDED REGION SIZE: 236

**rs369143448**

OLIGO startlentmgc%any_th3′_thhairpinseq

LEFT PRIMER 18 22 59.31 50.00 0.15 0.00 37.58 ATGTGACCTGCAACTCTCAGAG / ATGTGACCTGCAACTCTCAGAA

RIGHT PRIMER (CR) 120 20 58.67 50.00 0.00 0.00 0.00 TTGCCATGTGTAGATGTCCC

INTERNAL OLIGO 40 22 59.91 54.55 0.00 0.00 34.83 GACGTCTATCTGTGACTACCGG

SEQUENCE SIZE: 165

INCLUDED REGION SIZE: 165

**rs143353036**

OLIGO startlentmgc%any_th3’_thhairpinseq

LEFT PRIMER 13 19 59.17 52.63 0.00 0.00 47.04 TCACGGAGAAGCCACATGA / TCACGGAGAAGCCACATGG

RIGHT PRIMER (CR) 145 20 58.83 55.00 0.00 0.00 0.00 CAACGGAGATGCACGACTAG

INTERNAL OLIGO 56 20 60.03 55.00 0.00 0.00 0.00 TCTCTTGGTCAATGGCCTCC

SEQUENCE SIZE: 156

INCLUDED REGION SIZE: 156

**rs759369504**

OLIGO startlentmgc%any_th3′_thhairpinseq

LEFT PRIMER 3 24 58.82 45.83 10.70 10.70 40.84 CTGTAGATTTGCTTACCTCAGACG / CTGTAGATTTGCTTACCTCAGACT

RIGHT PRIMER (CR) 121 20 57.08 50.00 0.00 0.00 0.00 TCACCAAGATGAGCGATAGC

INTERNAL OLIGO 51 26 57.11 26.92 0.00 0.00 0.00 ATTGATTAATGGTTGTTATTTACGCT

SEQUENCE SIZE: 152

INCLUDED REGION SIZE: 152

FORWARD WILD PRIMER

FORWARD MUTANT PRIMER

COMMON REVERSE

PROB

The gene regions rs781673405, rs1244378045, rs767450259, rs750556128, rs369143448, rs143353036, and rs759369504 of TRAIL were evaluated for polymorphism using the RT-qPCR (Biorad cfx96) method (Table 1).

**Table 1:** RT-qPCR protocol used for rs781673405, rs1244378045, rs767450259, rs750556128, rs369143448, rs143353036 and rs759369504 gene regions

Reagent	Volume
2xRT-qPCR Mix	5 µL
Forward Primer	0025 µL
Reverse Primer	0025 µL
Sybr Green	006 µL
H_2_O	289 µL
DNA	2 µL
Total	10 µL

### Statistical analysis

All statistical analyses were conducted using the SPSS (Statistical Package for Social Sciences for Windows) 29.0 software, and a p-value of ≤ 0.05 was considered significant. The 2 × 2 Chi-Square test was used to compare the differences and significance of gene and allele distributions in the study groups. ANOVA test was used for inter-group statistical analysis of the parameters used in the study.

## Results

In our study, the genotype and allele distributions of the single nucleotide polymorphisms rs781673405, rs1244378045, rs767450259, rs750556128, rs369143448, rs143353036, and rs759369504 in the TRAIL gene were compared among diabetic obese, non-diabetic obese, and control groups.

In the analysis made with biochemical parameters, it was determined that there was a high degree of significance in the diabetic obese group when BMI, urea, glucose level, triglyceride, creatinine and HbA1c values were compared with the non-diabetic obese group and the control group. LDH and HDL values were found to be higher and significant in the non-diabetic obese group compared to the diabetic obese and control groups (*p* < 0.05) (Table 2).

**Table 2:** Biochemical parameters of the diabetic obese, non-diabetic obese and control groups

	Diabetic Obese *n* = 80			Non-diabetic Obese *n* = 80			Control *n* = 80			*p*
	Min	Max	Mean ± SD*	Min	Max	Mean ± SD*	Min	Max	Mean ± SD*	
Glucose	48	422	187.73 ± 75.19	75	105	89.95 ± 8.55	72	111	91.88 ± 8.07	***<0.001***
Urea	16	105	35.44 ± 16.74	13	37	24.16 ± 8.11	12	47	26.78 ± 7.66	***<0.001***
Creatinine	0.4	2.66	0.86 ± 0.35	0.34	0.9	0.60 ± 0.17	0.38	1.07	0.68 ± 0.15	***<0.001***
LDH	133	298	187.48 ± 32.80	138	330	188.05 ± 44.07	118	263	173.06 ± 30.58	***0.048***
T_Chol	112	301	203.26 ± 42.78	145	333	207.18 ± 47.48	96	359	209.38 ± 51.43	*0.79*
Trig	50	815	195.19 ± 129.79	29	936	132.27 ± 184.63	31	364	112.74 ± 62.56	***<0.001***
HDL	23	66.5	44.04 ± 9.94	20	111	54.68 ± 17.32	32	80	54.16 ± 11.27	***<0.001***
LDL	58	193	121.40 ± 32.90	54	242	130.36 ± 41.07	52	256	132.26 ± 42.69	*0.396*
HbA1c	5.1	13.7	8.56 ± 1.97	4.5	6	5.36 ± 0.36	4.6	6	5.33 ± 0.30	***<0.001***
BMI	30.04	45.79	35.17 ± 3.99	30.08	65.33	37.91 ± 8.08	18.34	29.97	25.01 ± 3.03	***<0.001***

Bold: statistically significant results

It was shown that the genotype distribution of the TRAIL rs767450259 polymorphism was statistically significant in diabetic obese individuals compared to the control group (*p* = 0.045). The T allele of the rs767450259 polymorphism was found to be significantly higher in the control group compared to the diabetic obese group (50 vs. 63.5% respectively; *p* = 0.016). The G allele of the rs369143448 single nucleotide polymorphism was found to be higher in the control group compared to the diabetic obese group (63.8 vs. 70.2% respectively; *p* = 0.044). However, no statistically significant difference was found between the genotype distributions (*p* > 0.05). In the rs750556128 single nucleotide polymorphism, the A allele was found to be significantly higher in the diabetic obese group compared to the control group (57.7 vs. 69% respectively; *p* = 0.01). Additionally, it was observed that having the A allele in the rs750556128 polymorphism of the TRAIL gene could increase the risk of diabetes by 1.3 times in obese individuals (OR: 1.37). However, no significant difference was found between the genotype distributions (*p* > 0.05).

**Fig. 1:**
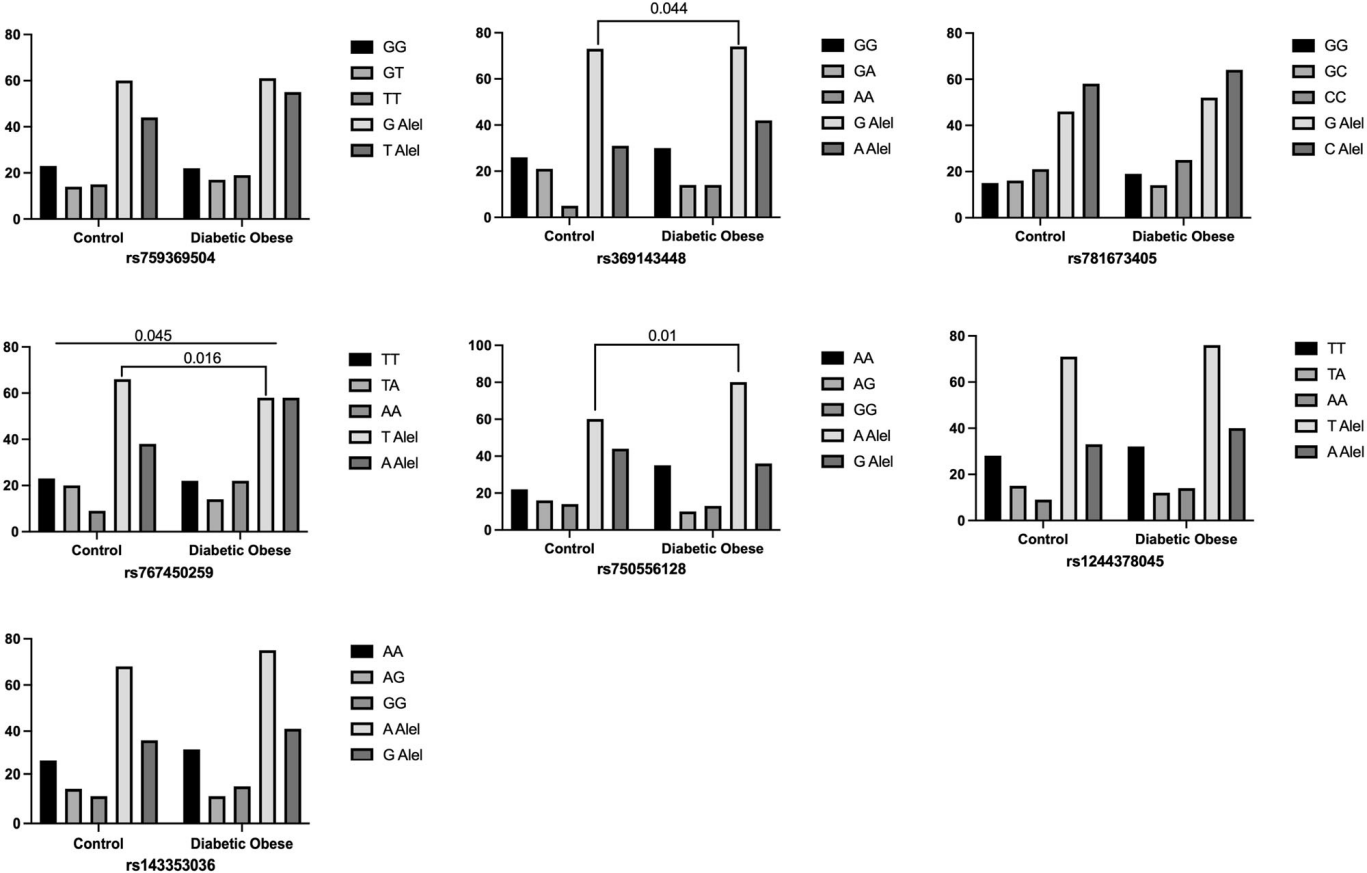
Comparison of genotype and allele distributions of rs781673405, rs1244378045, rs767450259, rs750556128, rs369143448, rs143353036 and rs759369504 polymorphisms in the TRAIL gene in diabetic obese subjects

In our study, we found that the single nucleotide polymorphisms rs781673405, rs1244378045, rs143353036, and rs759369504 of the TRAIL gene did not show any significant difference in genotype and allele distributions in diabetic obesity (*p* > 0.05) (Fig. 1).

In our study, when comparing the genotype and allele distributions of the rs781673405, rs1244378045, rs767450259, rs750556128, rs369143448, rs143353036, and rs759369504 single nucleotide polymorphisms of the TRAIL gene between non-diabetic obese and control groups, no statistically significant differences were found (*p* > 0.05) (Table 3).

**Table 3:** Comparison of genotype and allele distributions of rs781673405, rs1244378045, rs767450259, rs750556128, rs369143448, rs143353036, and rs759369504 polymorphisms in the TRAIL gene in non-diabetic obese individuals

rs	Genotype and Allel	Control	Non-Diabetic Obese	*P*	CI 95%
rs759369504	GG	23 (44.3%)	17 (43.6%)	0,991	
	GT	14 (26.9%)	11 (28.2%)		
	TT	15 (28.8%)	11 (28.2%)		
	G Allel	60 (52.6%)	45 (57.7%)	0,947	0.411–2.58
	T Allel	44 (47.4%)	33 (42.3%)	0,951	0.445–2.37
rs369143448	GG	26 (50%)	18 (46.2%)	0,87	
	GA	21 (40.4%)	16 (41%)		
	AA	5 (9.6%)	5 (12.8%)		
	G Allel	73 (70.2%)	52 (66.7%)	0,629	0.194–2.697
	A Allel	31 (29.8%)	26 (33.3%)	0,716	0.508–2.680
rs781673405	GG	15 (28.8%)	13 (33.3%)	0,877	
	GC	16 (30.8%)	12 (30.8%)		
	CC	21 (40.4%)	14 (35.9%)		
	G Allel	46 (44.2%)	38 (48.7%)	0,663	0.513–2.851
	C Allel	58 (55.8%)	40 (51.2%)	0,646	0.331–1.987
rs767450259	TT	23 (44.2%)	17 (43.6%)	0,572	
	TA	20 (38.5%)	12 (30.8%)		
	AA	9 (17.3%)	10 (25.6%)		
	T Allel	66 (63.5%)	46 (59%)	0,333	0.22–1.677
	A Allel	38 (36.5%)	32 (41%)	0,951	0.445–2.37
rs750556128	AA	22 (42.3%)	16 (41%)	0,917	
	AG	16 (30.8%)	11 (28.2%)		
	GG	14 (26.9%)	12 (30.8%)		
	A Allel	60 (57.7%)	43 (55%)	0,494	0.248–1.964
	G Allel	44 (42.3%)	35 (45%)	0,951	0.445–2.370
rs1244378045	TT	28 (53.8%)	20 (51.3%)	0,591	
	TA	15 (28.8%)	9 (23.1%)		
	AA	9 (17.3%)	10 (25.6%)		
	T Allel	71 (68.3%)	49 (62.8%)	0,333	0.22–1.677
	A Allel	33 (31.7%)	29 (37.2%)	0,808	0.483–2.546
rs143353036	AA	27 (51.9%)	21 (53.8%)	0,768	
	AG	14 (26.9%)	12 (30.8%)		
	GG	11 (21.2%)	6 (15.4%)		
	A Allel	68 (65.4%)	56 (71.8%)	0,485	0.494–4.412
	G Allel	36 (34.6%)	24 (28.2%)	0,856	0.403–2.127

No significant differences were found in the genotype and allele distributions of the rs781673405, rs1244378045, rs767450259, rs750556128, rs369143448, rs143353036, and rs759369504 single nucleotide polymorphisms of the TRAIL gene between the diabetic obese and non-diabetic obese groups (*p* > 0.05) (Table 4).

**Table 4:** Comparison of genotype and allele distributions of rs781673405, rs1244378045, rs767450259, rs750556128, rs369143448, rs143353036, and rs759369504 polymorphisms in the TRAIL gene between diabetic obese and non-diabetic obese groups

rs	Genotype and Allel	Diabetic Obese	Non-Diabetic Obese	*P*	CI 95%
rs759369504	GG	22 (37.9%)	17 (43.6%)	0,839	
	GT	17 (29.3%)	11 (28.2%)		
	TT	19 (32.8%)	11 (28.2%)		
	G Allel	61 (52.5%)	45 (57.7%)	0,634	0.511–3.011
	T Allel	55 (47.4%)	33 (42.3%)	0,577	0.346–1.8
rs369143448	GG	30 (51.7%)	18 (46.2%)	0,148	
	GA	14 (24.1%)	16 (41%)		
	AA	14 (24.2%)	5 (12.8%)		
	G Allel	74 (63.8%)	52 (66.7%)	0,168	0.710–6.597
	A Allel	42 (36.2%)	26 (33.3%)	0,591	0.554–2.819
rs781673405	GG	19 (32.8%)	13 (33.3%)	0,71	
	GC	14 (24.1%)	12 (30.8%)		
	CC	25 (43.1%)	14 (35.9%)		
	G Allel	52 (47.4%)	38 (48.7%)	0,478	0.587–3.120
	C Allel	64 (52.6%)	40 (51.2%)	0,953	0.411–2.308
rs767450259	TT	22 (38%)	17 (43.6%)	0,441	
	TA	14 (24.%)	12 (30.8%)		
	AA	22 (38%)	10 (25.6%)		
	T Allel	58 (50%)	46 (59%)	0,207	0.725–4.33
	A Allel	58 (50%)	32 (41%)	0,577	0.346–1.806
rs750556128	AA	35 (60.3%)	16 (41%)	0,167	
	AG	10 (17.2%)	11 (28.2%)		
	GG	13 (22.4%)	12 (30.8%)		
	A Allel	80 (69%)	43 (55%)	0,089	0.880–5.452
	G Allel	36 (31%)	35 (45%)	0,465	0.321–1.683
rs1244378045	TT	32 (55.2%)	20 (51.3%)	0,928	
	TA	12 (20.7%)	9 (23.1%)		
	AA	14 (24.1%)	10 (25.6%)		
	T Allel	76 (65.5%)	49 (62.8%)	0,866	0.361–2.356
	A Allel	40 (34.5%)	29 (37.2%)	0,706	0.518–2.638
rs143353036	AA	32 (55.2%)	21 (53.8%)	0,278	
	AG	11 (19%)	12 (30.8%)		
	GG	15 (25.8%)	6 (15.4%)		
	A Allel	75 (64.6%)	56 (71.8%)	0,219	0.672–5.482
	G Allel	41 (35.4%)	24 (28.2%)	0,898	0.467–2.383

In the diabetic obese group, each of the genetic regions listed in Table 5 was individually examined in terms of biochemical parameters. The findings obtained are as follows.

**Table 5:** Comparison of genotype and allele distributions of rs781673405, rs1244378045, rs767450259, rs750556128, rs369143448, rs143353036, and rs759369504 polymorphisms in the TRAIL gene in the diabetic obese group in terms of biochemical parameters

rs	Genotype and Allele		Age	Glucose	Urea	Creatinine	LDH	ALT	AST	T_Chol	Trig	HDL	LDL	Hba1c	BMI
rs759369504	TT genotype	Mean ± SE	56,37 ± 1,94	188,3 ± 10,61	36,36 ± 3.28	0,86 ± 0,05	201,67 ± 7,61	21,78 ± 2,06	19,3 ± 1,29	207,17 ± 9,76	181,69 ± 24,65	46,32 ± 1,75	123,96 ± 7,14	8,53 ± 0,32	34,71 ± 0,78
	G allele	Mean ± SE	54,34 ± 1,3	187,43 ± 11,5	34,99 ± 2,39	0,86 ± 0,05	181,03 ± 5,68	27,27 ± 2,48	21,00 ± 1,39	201,38 ± 5,73	202,2 ± 18,22	42,85 ± 1,42	120,12 ± 4,34	8,57 ± 0,3	35,41 ± 0,55
	*P* value		0,188	0,481	0,370	0,492	**0,021**	0,075	0,214	0,295	0,254	0,071	0,315	0,460	0,231
	GG genotip	Mean ± SE	54,3 ± 1,99	196,73 ± 17,82	39,96 ± 3,61	0,97 ± 0,09	179,16 ± 5,81	23,95 ± 2,46	19,74 ± 1,25	192,85 ± 7,14	196,05 ± 16,04	41,60 ± 1,86	113,79 ± 5,96	8,75 ± 0,45	35,67 ± 0,74
	T allel	Mean ± SE	55,46 ± 1,26	182,32 ± 8,24	32,50 ± 2,04	0,80 ± 0,03	192,93 ± 6,74	26,29 ± 2,48	20,84 ± 1,46	209,23 ± 6,56	194,69 ± 21,24	45,45 ± 1,37	125,90 ± 4,70	8,45 ± 0,23	34,87 ± 0,56
	*P* value		0,303	0,234	**0,039**	**0,040**	0,078	0,266	0,302	0,057	0,482	**0,049**	0,058	0,279	0,194
rs369143448	AA genotype	Mean ± SE	57,94 ± 1,79	212,33 ± 21,97	41,39 ± 5,74	0,93 ± 0,08	193,36 ± 9,71	21,47 ± 2,28	20,23 ± 1,42	207,00 ± 8,94	162,80 ± 16,48	46,42 ± 2,22	126,77 ± 9,07	8,70 ± 0,53	34,22 ± 0,85
	G allel	Mean ± SE	54,18 ± 1,28	180,58 ± 8,68	33,73 ± 1,82	0,84 ± 0,05	185,73 ± 5,46	26,56 ± 2,21	20,48 ± 1,25	202,14 ± 5,91	204,75 ± 18,16	43,34 ± 1,29	119,90 ± 4,06	8,52 ± 0,24	35,45 ± 0,52
	*P* value		0,073	0,058	**0,048**	0,188	0,252	0,120	0,461	0,342	0,115	0,125	0,225	0,365	0,127
	GG genotype	Mean ± SE	52,82 ± 1,78	173,67 ± 10,18	32,57 ± 2,35	0,87 ± 0,07	182,35 ± 7,24	28,17 ± 3,05	22,11 ± 1,88	197,25 ± 6,79	200,91 ± 24,94	43,72 ± 1,73	116,66 ± 4,77	8,39 ± 0,28	34,22 ± 0,47
	A allele	Mean ± SE	57,12 ± 1,18	201,10 ± 13,02	38,17 ± 2,97	0,85 ± 0,04	192,20 ± 6,17	22,79 ± 1,92	18,86 ± 0,84	208,95 ± 7,20	189,89 ± 16,32	44,33 ± 1,46	125,90 ± 5,64	8,71 ± 0,34	36,07 ± 0,72
	*P* value		**0,023**	0,052	0,073	0,376	0,152	0,068	0,061	0,121	0,354	0,394	0,109	0,240	**0,018**
rs781673405	CC genotype	Mean ± SE	55,88 ± 1,62	215,94 ± 12,83	34,95 ± 3,25	0,83 ± 0,05	197,53 ± 8,52	27,42 ± 3,09	22,12 ± 2,08	207,87 ± 9,43	224,54 ± 29,27	44,41 ± 1,65	119,55 ± 6,06	9,06 ± 0,36	35,21 ± 0,73
	G allele	Mean ± SE	54,39 ± 1,45	166,87 ± 10,19	35,82 ± 2,34	0,88 ± 0,06	180,90 ± 5,27	23,93 ± 2,14	19,20 ± 0,88	200,11 ± 5,40	173,02 ± 12,34	43,76 ± 1,53	122,76 ± 4,74	8,18 ± 0,27	35,14 ± 0,57
	*P* value		0,249	**0,002**	0,413	0,256	**0,043**	0,170	0,079	0,239	0,056	0,388	0,337	**0,024**	0,470
rs1244378045	AA genotype	Mean ± SE	57,32 ± 1,88	186,05 ± 16,63	33,98 ± 3,91	0,76 ± 0,04	211,33 ± 10,86	20,52 ± 2,21	17,65 ± 1,14	215,73 ± 10,03	200,20 ± 32,32	47,61 ± 1,97	131,96 ± 8,08	8,51 ± 0,42	36,04 ± 0,99
	T allele	Mean ± SE	54,16 ± 1,30	188,36 ± 9,82	35,97 ± 2,22	0,90 ± 0,05	179,53 ± 4,53	27,27 ± 2,30	21,49 ± 1,32	197,98 ± 5,57	193,26 ± 16,13	42,66 ± 1,32	117,25 ± 4,02	8,58 ± 0,26	34,84 ± 0,49
	*P* value		0,096	0,451	0,326	0,060	**0,001**	**0,047**	**0,045**	**0,052**	0,416	**0,023**	**0,038**	0,447	0,117
	TT genotype	Mean ± SE	54,73 ± 1,40	196,63 ± 11,91	35,95 ± 2,74	0,90 ± 0,06	178,62 ± 5,48	30,04 ± 2,97	23,02 ± 1,70	204,46 ± 6,95	203,60 ± 20,56	43,24 ± 1,49	120,48 ± 4,91	8,63 ± 0,32	34,75 ± 0,56
	A allele	Mean ± SE	55,33 ± 1,67	178,36 ± 11,83	34,91 ± 2,72	0,82 ± 0,04	197,96 ± 7,58	20,55 ± 1,68	17,61 ± 0,87	202,05 ± 7,21	186,12 ± 20,91	44,90 ± 1,69	122,37 ± 5,70	8,48 ± 0,31	35,61 ± 0,70
	*P* value		0,391	0,140	0,395	0,138	**0,020**	**0,004**	**0,003**	0,405	0,277	0,230	0,401	0,368	0,169
rs143353036	AA genotype	Mean ± SE	55,30 ± 1,41	188,51 ± 12,52	35,26 ± 2,28	0,89 ± 0,05	176,68 ± 5,10	28,43 ± 2,79	22,52 ± 1,67	201,43 ± 5,65	187,13 ± 17,20	44,24 ± 1,38	122,72 ± 4,82	8,46 ± 0,32	34,90 ± 0,55
	G allele	Mean ± SE	54,70 ± 1,68	186,81 ± 11,09	35,64 ± 3,20	0,83 ± 0,06	202,60 ± 7,79	21,92 ± 2,04	17,92 ± 0,84	205,41 ± 8,63	204,83 ± 24,78	43,79 ± 1,83	119,77 ± 5,89	8,67 ± 0,31	35,49 ± 0,72
	*P* value		0,392	0,460	0,461	0,250	**0,003**	**0,035**	**0,012**	0,350	0,275	0,421	0,348	0,319	0,254
rs767450259	TT genotype	Mean ± SE	55,48 ± 1,44	186,87 ± 13,71	38,33 ± 3,42	0,98 ± 0,09	178,89 ± 5,09	27,63 ± 2,09	21,47 ± 1,06	196,04 ± 8,20	200,34 ± 20,63	43,80 ± 1,91	113,87 ± 5,99	8,59 ± 0,38	35,13 ± 0,66
	A allele	Mean ± SE	54,74 ± 1,52	188,27 ± 10,75	33,66 ± 2,27	0,79 ± 0,03	192,63 ± 6,82	24,01 ± 2,62	19,78 ± 1,51	207,65 ± 6,23	191,87 ± 20,16	44,19 ± 1,38	126,10 ± 4,68	8,54 ± 0,27	35,19 ± 0,60
	*P* value		0,369	0,468	0,120	**0,025**	0,057	0,165	0,212	0,130	0,390	0,433	0,055	0,452	0,474
rs750556128	GG genotype	Mean ± SE	53,43 ± 1,78	191,79 ± 16,75	30,92 ± 1,58	0,76 ± 0,03	188,44 ± 8,48	23,41 ± 2,67	19,19 ± 1,09	210,96 ± 8,27	196,72 ± 32,77	44,28 ± 1,75	129,85 ± 5,92	8,64 ± 0,39	35,52 ± 0,83
	A allele	Mean ± SE	55,89 ± 1,35	185,54 ± 9,39	37,93 ± 2,80	0,92 ± 0,06	186,90 ± 5,73	26,49 ± 2,37	21,10 ± 1,45	199,08 ± 6,20	194,40 ± 14,53	43,91 ± 1,45	116,92 ± 4,68	8,51 ± 0,27	34,98 ± 0,53
	*P* value		0,140	0,363	**0,016**	**0,006**	0,438	0,208	0,186	0,128	0,470	0,439	**0,049**	0,394	0,286
	AA genotype	Mean ± SE	54,80 ± 1,54	181,50 ± 11,89	38,34 ± 3,34	0,94 ± 0,08	185,38 ± 6,67	26,25 ± 2,27	19,76 ± 0,98	191,52 ± 7,93	168,51 ± 14,19	44,13 ± 2,01	113,17 ± 5,45	8,29 ± 0,36	34,65 ± 0,60
	G allele	Mean ± SE	55,16 ± 1,47	191,46 ± 11,46	33,75 ± 2,33	0,82 ± 0,04	188,53 ± 6,33	24,91 ± 2,55	20,80 ± 1,51	210,00 ± 6,22	211,52 ± 21,67	43,98 ± 1,33	126,27 ± 4,89	8,72 ± 0,28	35,49 ± 0,62
	*P* value		0,436	0,285	0,126	0,071	0,379	0,360	0,312	**0,037**	**0,050**	0,475	**0,045**	0,176	0,183

Bold: statistically significant results

The analysis of rs759369504;

- In diabetic obese individuals, it was reported that those with the TT genotype had higher LDH levels compared to those with the G allele, and this difference was statistically significant (*p* = 0.021). This result suggests that the TT genotype may increase LDH levels, and this increase is statistically significant. Since LDH is an indicator of tissue damage, it is possible that this genotype may be associated with tissue damage or cell breakdown in diabetic obese individuals.
- In diabetic obese individuals, those with the GG genotype were found to have higher urea and creatinine levels compared to those carrying the T allele, and this difference was statistically significant (*p* = 0.039; *p* = 0.04, respectively). This suggests that the GG genotype may have an impact on kidney function. Elevated urea and creatinine levels are indicators of impaired kidney function.
- In diabetic obese individuals, the HDL level in those carrying the T allele was found to be higher and statistically significant compared to those with the GG genotype (*p* = 0.049). The higher HDL levels in individuals carrying the T allele suggest that this allele may positively influence the lipid profile and potentially reduce cardiovascular risks.
- In the rs759369504 single nucleotide polymorphism, no significant differences were found in genotype and allele distributions when evaluated in terms of age, glucose, ALT, AST, total cholesterol, triglycerides, HbA1c, and BMI (*p* > 0.05).

The analysis of rs369143448;

- In diabetic obese individuals with the AA genotype, urea levels were found to be higher and statistically significant compared to those carrying the G allele (*p* = 0.048). The elevated urea levels in individuals with the AA genotype suggest that this genotype may negatively impact kidney function.
- Diabetic obese individuals carrying the A allele were found to have higher and statistically significant age, glucose levels, and BMI compared to those with the GG genotype (*p* = 0.023, *p* = 0.052, and *p* = 0.018, respectively).
- In the rs369143448 single nucleotide polymorphism, no significant differences were found in genotype and allele distributions when evaluated in terms of glucose, LDH, ALT, AST, total cholesterol, triglycerides, HDL, LDL, and HbA1c (*p* > 0.05). The higher age, glucose, and BMI values in individuals carrying the A allele suggest that this allele may be associated with aging, blood sugar control, and obesity in diabetic obese individuals, and these associations are statistically significant.

The analysis of rs781673405;

- In diabetic obese individuals with the CC genotype, glucose, LDH, and HbA1c levels were found to be higher and statistically significant compared to those carrying the G allele (*p* = 0.002, *p* = 0.043, and *p* = 0.024, respectively). The elevated glucose, LDH, and HbA1c levels in individuals with the CC genotype suggest that this genotype may be associated with the progression of diabetes and tissue damage.
- In the rs781673405 single nucleotide polymorphism, no significant differences were found in genotype and allele distributions when evaluated in terms of age, urea, ALT, AST, total cholesterol, triglycerides, HDL, LDL, and BMI (*p* > 0.05).

The analysis of rs12443788045;

- In diabetic obese individuals with the AA genotype, LDH, total cholesterol, HDL, and LDL levels were found to be higher and statistically significant compared to those carrying the T allele (*p* = 0.001, *p* = 0.052, *p* = 0.023, and *p* = 0.038, respectively). The elevated levels of these parameters in individuals with the AA genotype suggest that this genotype may negatively impact the lipid profile and tissue damage.
- In diabetic obese individuals carrying the T allele, ALT and AST levels were found to be higher and statistically significant compared to those with the AA genotype (*p* = 0.047 and *p* = 0.045, respectively). These findings suggest that the T allele may have negative effects on liver function.
- In diabetic obese individuals carrying the A allele, LDH levels were found to be higher and statistically significant compared to those with the TT genotype (*p* = 0.02).
- Diabetic obese individuals with the TT genotype were found to have higher ALT and AST levels compared to those carrying the A allele (*p* = 0.004 and *p* = 0.003, respectively).
- In the rs12443788045 single nucleotide polymorphism, no significant differences were found in genotype and allele distributions when evaluated in terms of age, glucose, urea, triglycerides, HbA1c, and BMI (*p* > 0.05).

The analysis of rs143353036;

- In diabetic obese individuals, those carrying the G allele were found to have higher and statistically significant LDH levels compared to those with the AA genotype (*p* = 0.003). The elevated LDH levels in individuals with the G allele suggest that this allele may be associated with tissue damage.
- In the diabetic obese group, individuals with the AA genotype had higher ALT and AST levels compared to those carrying the G allele, and these differences were statistically significant (*p* = 0.035; *p* = 0.012). The elevated ALT and AST levels in individuals with the AA genotype suggest that this genotype may negatively affect liver function.
- In the rs143353036 single nucleotide polymorphism, no significant differences were found in genotype and allele distributions when evaluated in terms of age, glucose, urea, creatinine, total cholesterol, triglycerides, HDL, LDL, HbA1c, and BMI (*p* > 0.05).

The analysis of rs767450259;

- In diabetic obese individuals, those with the AA genotype were found to have higher and statistically significant urea and creatinine levels compared to those carrying the T allele (*p* = 0.016; *p* = 0.009). The elevated urea and creatinine levels in individuals with the AA genotype suggest that this genotype may negatively affect kidney function.
- Diabetic obese individuals with the TT genotype were found to have higher creatinine levels compared to those carrying the A allele, and this difference was statistically significant (*p* = 0.0262). The elevated creatinine levels in individuals with the TT genotype suggest that this genotype may have negative effects on kidney function.
- In diabetic obese individuals, those carrying the A allele were found to have higher LDL levels compared to those with the TT genotype, and this difference approached statistical significance (*p* = 0.055). The elevated LDL levels in individuals with the A allele suggest that this allele may negatively affect the lipid profile.
- In the rs767450259 single nucleotide polymorphism, no significant differences were found in genotype and allele distributions when evaluated in terms of age, glucose, LDH, ALT, total cholesterol, triglycerides, HDL, LDL, HbA1c, and BMI (p > 0.05).

The analysis of rs750556128;

- In diabetic obese individuals, those with the GG genotype were found to have higher and statistically significant urea and creatinine levels compared to those carrying the A allele (*p* = 0.016; *p* = 0.006, respectively).
- In diabetic obese individuals with the GG genotype, LDL levels were found to be higher and statistically significant compared to those carrying the A allele (*p* = 0.049).
- In diabetic obese individuals carrying the G allele, triglyceride, LDL levels and total cholesterol levels were found to be higher and statistically significant compared to those with the AA genotype (*p* = 0.05, *p* = 0.045 and 0037 respectively). The elevated urea and creatinine levels in individuals with the GG genotype suggest that this genotype may negatively affect kidney function, while the high LDL levels indicate a potential negative impact on the lipid profile. Particularly, the high triglyceride and LDL levels in individuals carrying the G allele suggest that G allele carriage may contribute to obesity in diabetic individuals.
- In the rs750556128 single nucleotide polymorphism, no significant differences were found in genotype and allele distributions when evaluated in terms of age, glucose, LDH, ALT, AST, total cholesterol, HDL, HbA1c, and BMI (*p* > 0.05).

In the non-diabetic obese group, each of the rs variants listed in Table 6 was individually examined in terms of biochemical parameters. The findings obtained are as follows.

**Table 6:** Comparison of genotype and allele distributions of rs781673405, rs1244378045, rs767450259, rs750556128, rs369143448, rs143353036, and rs759369504 polymorphisms in the TRAIL gene in the non-diabetic obese group in terms of biochemical parameters

rs	Genotype and allele	Age		Glucose	Urea	Creatinine	LDH	ALT	AST	T_Chol	Trig	HDL	LDL	Hba1c	BMI
rs759369504	TT genotype	Mean ± SE	40,50 ± 4,69	99,85 ± 5,29	21,60 ± 4,09	0,70 ± 0,05	166,33 ± 22,36	47,54 ± 13,21	29,67 ± 8,02	191,83 ± 20,93	118,94 ± 15,28	48,38 ± 4,30	120,67 ± 12,10	5,31 ± 0,10	38,42 ± 1,77
	G allele	Mean ± SE	43,36 ± 2,40	91,90 ± 2,28	25,59 ± 1,88	0,66 ± 0,03	188,89 ± 10,33	22,07 ± 2,12	20,95 ± 1,07	200,90 ± 8,95	132,53 ± 32,21	51,06 ± 3,01	121,86 ± 7,06	5,37 ± 0,07	37,71 ± 1,48
	*P* value	0,28		0,058	0,19	0,264	0,207	**0,042**	0,154	0,34	0,406	0,327	0,467	0,324	0,396
	GG genotip	Mean ± SE	37,00 ± 1,78	107,25 ± 10,44	13,00 ± 0,10	0,70 ± 0,06	146,00 ± 0,10	23,25 ± 8,50	21,13 ± 2,16	153,33 ± 13,28	129,63 ± 5,27	38,88 ± 4,72	96,50 ± 6,38	5,55 ± 0,29	43,07 ± 4,66
	T allel	Mean ± SE	43,15 ± 2,33	92,57 ± 2,12	25,31 ± 1,71	0,66 ± 0,03	187,65 ± 9,64	29,13 ± 4,34	23,23 ± 2,30	203,66 ± 8,44	129,08 ± 27,43	51,78 ± 2,65	124,53 ± 6,52	5,34 ± 0,06	37,39 ± 1,19
	*P* value	**0,025**		**0,026**	0,092	0,359	0,179	0,337	0,386	**0,041**	0,497	0,057	0,078	0,142	0,085
rs369143448	GG genotype	Mean ± SE	44,04 ± 3,27	95,80 ± 3,07	26,44 ± 2,55	0,69 ± 0,04	177,78 ± 10,56	29,83 ± 6,34	24,17 ± 3,82	208,61 ± 12,02	99,59 ± 11,99	53,56 ± 2,23	129,42 ± 10,04	5,42 ± 0,08	35,79 ± 1,04
	A allele	Mean ± SE	40,80 ± 2,56	91,79 ± 3,16	22,79 ± 2,01	0,64 ± 0,04	191,58 ± 14,56	27,16 ± 4,74	21,78 ± 1,36	189,53 ± 10,71	155,57 ± 44,57	47,29 ± 4,41	113,74 ± 6,45	5,29 ± 0,09	40,54 ± 2,18
	*P* value	0,22		0,185	0,149	0,156	0,24	0,372	0,287	0,123	0,128	0,107	0,099	0,14	**0,03**
	CC genotype	Mean ± SE	39,78 ± 3,63	96,65 ± 3,74	23,43 ± 2,77	0,75 ± 0,03	175,20 ± 15,44	39,54 ± 8,45	27,43 ± 5,09	200,33 ± 16,98	108,53 ± 10,70	48,34 ± 2,83	129,27 ± 11,58	5,31 ± 0,07	38,47 ± 1,77
	G allele	Mean ± SE	44,48 ± 2,61	92,15 ± 2,70	25,68 ± 2,19	0,62 ± 0,04	188,94 ± 11,46	21,42 ± 3,08	20,32 ± 1,11	198,83 ± 8,95	143,86 ± 41,16	51,78 ± 3,70	116,57 ± 6,50	5,39 ± 0,09	37,52 ± 1,58
	*P* value	0,143		0,162	0,269	**0,01**	0,273	**0,029**	0,095	0,466	0,241	0,254	0,155	0,243	0,348
rs781673405	CC genotype	Mean ± SE	39,78 ± 3,63	96,65 ± 3,74	23,43 ± 2,77	0,75 ± 0,03	175,20 ± 15,44	39,54 ± 8,45	27,43 ± 5,09	200,33 ± 16,98	108,53 ± 10,70	48,34 ± 2,83	129,27 ± 11,58	5,31 ± 0,07	38,47 ± 1,77
	G allele	Mean ± SE	44,48 ± 2,61	92,15 ± 2,70	25,68 ± 2,19	0,62 ± 0,04	188,94 ± 11,46	21,42 ± 3,08	20,32 ± 1,11	198,83 ± 8,95	143,86 ± 41,16	51,78 ± 3,70	116,57 ± 6,50	5,39 ± 0,09	37,52 ± 1,58
	*P* value	0,143		0,162	0,269	**0,01**	0,273	**0,029**	0,095	0,466	0,241	0,254	0,155	0,243	0,348
	GG genotype	Mean ± SE	45,47 ± 4,23	90,33 ± 2,15	28,04 ± 2,80	0,65 ± 0,06	190,09 ± 16,27	22,60 ± 5,06	19,67 ± 1,50	194,92 ± 10,91	78,64 ± 13,21	55,00 ± 5,79	122,00 ± 10,09	5,28 ± 0,09	34,32 ± 1,21
	C allele	Mean ± SE	41,17 ± 2,42	95,86 ± 3,14	22,50 ± 1,99	0,68 ± 0,03	180,80 ± 9,07	31,79 ± 5,49	24,90 ± 3,11	201,96 ± 11,36	151,36 ± 33,83	48,04 ± 2,27	121,36 ± 7,66	5,39 ± 0,08	39,69 ± 1,56
	*P* value	0,174		0,077	**0,05**	0,336	0,317	0,14	0,117	0,341	0,086	0,095	0,48	0,209	**0,015**
rs750556128	GG genotype	Mean ± SE	43,50 ± 3,54	95,38 ± 4,65	21,70 ± 3,18	0,59 ± 0,06	197,40 ± 34,03	18,71 ± 2,22	17,54 ± 0,66	179,67 ± 7,49	107,33 ± 25,37	55,14 ± 10,48	111,57 ± 11,77	5,36 ± 0,14	36,31 ± 2,20
	A allele	Mean ± SE	42,41 ± 2,50	93,60 ± 2,51	25,57 ± 1,95	0,68 ± 0,03	182,00 ± 7,33	30,84 ± 4,83	24,32 ± 2,53	203,41 ± 9,55	133,50 ± 28,84	49,36 ± 2,06	123,84 ± 6,91	5,36 ± 0,07	38,24 ± 1,35
	*P* value	0,424		0,379	0,197	0,097	0,34	0,122	0,103	**0,031**	0,347	0,188	0,219	0,483	0,267
	AA genotype	Mean ± SE	45,37 ± 3,25	89,88 ± 2,13	27,64 ± 2,56	0,67 ± 0,04	183,78 ± 9,39	32,13 ± 7,74	27,79 ± 4,52	213,47 ± 14,44	159,77 ± 56,10	49,84 ± 2,48	129,56 ± 10,15	5,34 ± 0,10	37,40 ± 1,98
	G allele	Mean ± SE	40,58 ± 2,82	96,58 ± 3,29	23,09 ± 2,21	0,66 ± 0,04	187,08 ± 15,22	26,27 ± 4,35	19,79 ± 1,47	188,75 ± 8,82	107,26 ± 12,09	50,85 ± 3,96	115,77 ± 7,29	5,37 ± 0,07	38,26 ± 1,45
	*P* value	0,137		0,069	0,099	0,451	0,433	0,24	**0,029**	0,067	0,147	0,423	0,132	0,426	0,361
rs1244378045	AA genotype	Mean ± SE	46,29 ± 3,73	102,29 ± 9,21	21,33 ± 2,40	0,60 ± 0,03	169,75 ± 12,52	21,56 ± 3,68	22,61 ± 2,75	181,67 ± 22,91	254,17 ± 138,22	42,98 ± 5,92	107,17 ± 13,18	5,46 ± 0,15	39,96 ± 3,17
	T allele	Mean ± SE	41,92 ± 2,44	92,31 ± 1,91	25,30 ± 1,88	0,68 ± 0,03	189,41 ± 11,15	29,95 ± 4,71	23,11 ± 2,46	203,00 ± 8,64	104,13 ± 8,36	51,82 ± 2,71	124,28 ± 6,69	5,34 ± 0,06	37,52 ± 1,27
	*P* value	0,233		0,163	0,241	**0,041**	0,212	0,223	0,465	0,165	0,164	0,1	0,153	0,222	0,228
	TT genotype	Mean ± SE	40,04 ± 3,02	91,72 ± 2,52	25,66 ± 2,31	0,70 ± 0,04	179,09 ± 9,57	30,92 ± 6,17	23,62 ± 3,46	203,29 ± 9,53	93,71 ± 7,98	50,27 ± 2,13	125,83 ± 6,47	5,27 ± 0,07	38,13 ± 1,75
	A allele	Mean ± SE	46,11 ± 2,81	97,00 ± 3,89	23,65 ± 2,54	0,62 ± 0,04	192,90 ± 16,96	25,34 ± 4,34	22,24 ± 1,70	193,43 ± 14,76	178,73 ± 55,91	50,66 ± 5,54	115,07 ± 11,71	5,48 ± 0,10	37,57 ± 1,46
	*P* value	0,082		0,121	0,287	0,069	0,238	0,249	0,374	0,28	**0,042**	0,474	0,195	**0,042**	0,408
rs143353036	GG genotype	Mean ± SE	38,64 ± 4,81	97,44 ± 5,44	24,00 ± 7,00	0,70 ± 0,06	156,00 ± 14,19	34,34 ± 7,97	24,35 ± 2,15	169,86 ± 15,49	113,49 ± 14,16	44,28 ± 3,11	112,11 ± 11,51	5,43 ± 0,14	39,92 ± 2,38
	A allele	Mean ± SE	43,88 ± 2,36	92,73 ± 2,31	24,98 ± 1,78	0,66 ± 0,03	190,61 ± 10,34	26,61 ± 4,65	22,56 ± 2,74	206,71 ± 8,98	133,61 ± 31,13	52,33 ± 3,06	124,52 ± 7,06	5,34 ± 0,07	37,24 ± 1,35
	*P* value	0,148		0,178	0,431	0,257	0,102	0,203	0,356	**0,034**	0,368	0,086	0,194	0,248	0,166
	AA genotype	Mean ± SE	42,57 ± 2,94	93,15 ± 2,69	24,53 ± 2,30	0,66 ± 0,04	197,17 ± 14,48	22,19 ± 3,23	18,91 ± 1,22	202,90 ± 9,01	93,74 ± 11,21	51,50 ± 4,25	124,30 ± 6,57	5,31 ± 0,07	36,56 ± 1,47
	G allele	Mean ± SE	42,64 ± 3,18	94,75 ± 3,58	25,33 ± 2,64	0,67 ± 0,04	170,33 ± 8,82	35,29 ± 7,28	27,14 ± 3,83	195,13 ± 14,48	168,70 ± 49,00	49,23 ± 2,43	118,56 ± 10,59	5,42 ± 0,10	39,30 ± 1,83
	*P* value	0,493		0,36	0,41	0,426	0,081	0,056	**0,02**	0,32	0,063	0,328	0,32	0,186	0,124
rs767450259	AA genotype	Mean ± SE	43,50 ± 3,54	95,38 ± 4,65	21,70 ± 3,18	0,59 ± 0,06	197,40 ± 34,03	18,71 ± 2,22	17,54 ± 0,66	179,67 ± 7,49	107,33 ± 25,37	55,14 ± 10,48	111,57 ± 11,77	5,36 ± 0,14	36,31 ± 2,20
	T allele	Mean ± SE	42,41 ± 2,50	93,60 ± 2,51	25,57 ± 1,95	0,68 ± 0,03	182,00 ± 7,33	30,84 ± 4,83	24,32 ± 2,53	203,41 ± 9,55	133,50 ± 28,84	49,36 ± 2,06	123,84 ± 6,91	5,36 ± 0,07	38,24 ± 1,35
	*P* value	0,424		0,379	0,197	0,097	0,34	0,122	0,103	**0,031**	0,347	0,188	0,219	0,483	0,267
	TT genotype	Mean ± SE	45,37 ± 3,25	89,88 ± 2,13	27,64 ± 2,56	0,67 ± 0,04	183,78 ± 9,39	32,13 ± 7,74	27,79 ± 4,52	213,47 ± 14,44	159,77 ± 56,10	49,84 ± 2,48	129,56 ± 10,15	5,34 ± 0,10	37,40 ± 1,98
	A allele	Mean ± SE	40,58 ± 2,82	96,58 ± 3,29	23,09 ± 2,21	0,66 ± 0,04	187,08 ± 15,22	26,27 ± 4,35	19,79 ± 1,47	188,75 ± 8,82	107,26 ± 12,09	50,85 ± 3,96	115,77 ± 7,29	5,37 ± 0,07	38,26 ± 1,45
	*P* value	0,137		0,069	0,099	0,451	0,433	0,24	**0,029**	0,067	0,147	0,423	0,132	0,426	0,361

Bold: statistically significant results

The analysis of rs759369504;

- In non-diabetic obese individuals, those with the TT genotype were found to have higher ALT levels compared to those carrying the G allele, and this difference was statistically significant (*p* = 0.042). This suggests that the TT genotype may increase ALT levels, and this increase is statistically significant. Since ALT levels are related to liver function, this genotype may affect liver health.
- When examining age, glucose, and total cholesterol levels, it was found that individuals carrying the T allele had higher and statistically significant age and total cholesterol levels compared to those with the GG genotype (*p* = 0.025; *p* = 0.041, respectively). However, glucose levels were higher and statistically significant in individuals with the GG genotype compared to those carrying the T allele (*p* = 0.026). The higher age and total cholesterol levels in those carrying the T allele suggest that this allele may influence the aging process and lipid profile. The elevated glucose levels in individuals with the GG genotype indicate that this genotype may negatively affect glucose metabolism.
- In the rs759369504 single nucleotide polymorphism, no significant differences were found in genotype and allele distributions when evaluated in terms of urea, creatinine, LDH, AST, triglycerides, HDL, LDL, HbA1c, and BMI (*p* > 0.05).

The analysis of rs369143448;

- In the non-diabetic obese group, individuals with the CC genotype were found to have higher and statistically significant creatinine and ALT levels compared to those carrying the G allele (*p* = 0.01; *p* = 0.029, respectively). This suggests that individuals with the CC genotype may have impaired kidney function and that their liver health may be negatively affected.
- In the rs369143448 single nucleotide polymorphism, no significant differences were found in genotype and allele distributions when evaluated in terms of age, glucose, urea, LDH, AST, total cholesterol, triglycerides, HDL, LDL, HbA1c, and BMI (*p* > 0.05).

The analysis of rs781673405;

- In the non-diabetic obese group, individuals with the CC genotype were found to have higher and statistically significant creatinine and ALT levels compared to those carrying the G allele (*p* = 0.01; *p* = 0.029, respectively). This suggests that individuals with the CC genotype may experience impaired kidney and liver function.
- Individuals with the GG genotype were also found to have higher and statistically significant urea levels compared to those carrying the C allele (*p* = 0.05). This suggests that individuals with the GG genotype may have impaired kidney function. The association of both GG and CC genotypes with kidney function indicates that the heterozygous genotype may also have a negative impact on kidney function.
- Individuals with the carrying C allele were also found to have higher and statistically significant urea levels compared to GG genotype (*p* = 0.015).
- In the rs3781673405 single nucleotide polymorphism, no significant differences were found in genotype and allele distributions when evaluated in terms of age, glucose, LDH, ALT, AST, total cholesterol, triglycerides, HDL, LDL and HbA1c (*p* > 0.05).

The analysis of rs750556128;

- In the non-diabetic obese group, individuals carrying the A allele were found to have higher and statistically significant total cholesterol levels compared to those with the GG genotype (*p* = 0.031). The elevated total cholesterol levels in individuals with the A allele suggest that this allele may negatively affect the lipid profile.
- In non-diabetic obese individuals, those with the AA genotype were found to have higher and statistically significant AST levels compared to those carrying the G allele (*p* = 0.029). The elevated AST levels in individuals with the AA genotype suggest that this genotype may negatively affect liver function.
- In the rs750556128 single nucleotide polymorphism, no significant differences were found in genotype and allele distributions when evaluated in terms of age, glucose, urea, creatinine, LDH, ALT, triglycerides, HDL, LDL, HbA1c, and BMI (*p* > 0.05).

The analysis of rs1244378045;

- In the non-diabetic obese group, individuals carrying the T allele were found to have higher and statistically significant creatinine levels compared to those with the AA genotype (*p* = 0.041). The elevated creatinine levels in individuals with the T allele suggest that this allele may negatively affect kidney function.
- In non-diabetic obese individuals, those carrying the A allele were found to have higher and statistically significant triglyceride and HbA1c levels compared to those with the TT genotype (*p* = 0.042; *p* = 0.042, respectively). The elevated triglyceride and HbA1c levels in individuals with the A allele suggest that this allele may negatively affect the lipid profile and glucose metabolism.
- In the rs1244378045 single nucleotide polymorphism, no significant differences were found in genotype and allele distributions when evaluated in terms of age, glucose, urea, LDH, ALT, AST, total cholesterol, HDL, LDL, and BMI (*p* > 0.05).

The analysis of rs143353036;

- In non-diabetic obese individuals, those carrying the A allele were found to have higher and statistically significant total cholesterol levels compared to those with the GG genotype (*p* = 0.034). The elevated total cholesterol levels in individuals with the A allele suggest that this allele may negatively affect the lipid profile.
- In the non-diabetic obese group, individuals carrying the G allele were found to have higher and statistically significant AST levels compared to those with the AA genotype (*p* = 0.02). The elevated AST levels in individuals with the G allele suggest that this allele may negatively affect liver function.
- In the rs143353036 single nucleotide polymorphism, no significant differences were found in genotype and allele distributions when evaluated in terms of age, glucose, urea, creatinine, LDH, ALT, HDL, LDL, HbA1c, and BMI (p > 0.05).

The analysis of rs767450259;

- In non-diabetic obese individuals, those carrying the T allele were found to have higher and statistically significant total cholesterol levels compared to those with the AA genotype (*p* = 0.031). The elevated total cholesterol levels in individuals with the T allele suggest that this allele may negatively affect the lipid profile.
- In the non-diabetic obese group, individuals with the TT genotype were found to have higher and statistically significant AST levels compared to those carrying the A allele (*p* = 0.029). The elevated AST levels in individuals with the TT genotype suggest that this genotype may negatively affect liver function.
- In the rs767450259 single nucleotide polymorphism, no significant differences were found in genotype and allele distributions when evaluated in terms of age, glucose, urea, creatinine, LDH, ALT, triglycerides, HDL, LDL, HbA1c, and BMI (*p* > 0.05).

## Discussion

While most previous studies on single nucleotide polymorphisms (SNPs) related to TRAIL have focused on cancer research, there are very few studies on diabetes, and no studies have been found related to obesity. According to earlier research datas, there is increasing evidence that TRAIL may play a protective role against diabetes [9, 11].

Polymorphism studies on TRAIL and TRAIL receptors in diabetic patients have reported that the genotypes identified may be candidates for prognostic evaluation of diabetes. The details of these studies are provided below.

It has been reported that the CC genotype in the 1595 C/T (rs1131580) region of the TRAIL gene, a SNP in non-alcoholic fatty liver disease, which is a risk factor for type II diabetes, is associated with type II diabetes and could be a candidate for the prognostic evaluation of type II diabetes [13]. Additionally, in patients with type II diabetes, the CC genotype and the frequency of the C allele in the 1595 region of TRAIL were found to be significantly higher compared to the control group. Accordingly, it has been reported that the CC genotype in the 1595 C/T (rs1131580) region of TRAIL is associated with type II diabetes and could be a candidate for evaluating type II diabetes [13]. In a research, a genomic DNA analysis was conducted on 73 pregnant women with gestational diabetes before diet therapy and 72 healthy pregnant women, examining the rs2073617 T950C polymorphism, which is a soluble receptor of TRAIL, Osteoprotegerin. A significant increase (OR = 3.63) in the distribution of the CT genotype was observed between the groups. Osteoprotegerin levels were reported to be high in women with gestational diabetes before diet therapy [15].

In addition to these studies, other research on SNPs related to TRAIL variants, which have been studied for the first time in different disease groups, is summarized below.

In a study on black women, the TRAIL rs13074711 variant was found to be associated with breast cancer, and it was reported that TNSF10 plays an important role in regulating antiviral immune responses in breast cancer [16]. A study conducted on multiple sclerosis patients in Spain investigated seven variants of TRAIL, including rs4894559, rs4872077, rs11779484, rs4460370, rs9314261, rs3924519, and rs1001793, and found that all variants were associated with the disease. Additionally, it was noted that the G, T, and A alleles of rs4894559, rs4872077, and rs1001793, respectively, were more strongly associated with multiple sclerosis, serving as protective factors [17]. Another study found that the TNSF10 rs9859259 variant, which plays a role in the apoptosis of human colon cancer cells, was associated with Crohn’s disease and could explain part of the high risk of colorectal cancer in these patients [18].

In breast cancer, the SNPs rs3815496, rs1131532, and rs1131535 of TRAIL were found to be associated with the radiosensitivity of T4EM lymphocytes, which determine the onset of acute and subacute dermatitis after radiotherapy. The radiosensitivity of T4EM lymphocytes could also be used to predict the response to radiotherapy [19]. Another cancer study found that the TNSF10 SNP rs6785617 was significantly associated with malignant tumors seen in low-grade ovarian cancer [20]. The SNPs rs3815496, rs3136597, and rs4894559 of TRAIL were found to be associated with prostate cancer, and the rs6497287 genetic variant was more strongly associated with aggressive prostate cancer. This significant finding strengthens the hypothesis of genetic susceptibility to prostate cancer [21]. A study in the Chinese population reported that the TNSF10 rs35975099 gene polymorphism does not play a significant role in migraine pathogenesis [22].

The number of people with obesity and diabetes in Turkey is increasing day by day. This situation is population-specific and varies in different populations depending on lifestyles, nutritional habits, environmental and genetic factors. This study is of particular importance in that it reflects the Turkish population and is the first study conducted with these variants in the Turkish population. As a result of our literature review, we found that studies on TRAIL variants in the Turkish population focus more on cancer research rather than obesity and diabetes. For example; Yildiz Y et al. Found that the genetic variants of TRAIL at position 1595 in exon 5 might be associated with progression of breast cancer [23]. Another study explained that TRAIL 1595 C allele may be used as a low-penetrant risk factor for bladder cancer development in a Turkish population [24].

In our study, the presence of mutations in rs781673405, rs1244378045, rs767450259, rs750556128, rs369143448, rs143353036, and rs759369504 was investigated for the first time. The results indicated that the genotype distribution of the TRAIL rs767450259 polymorphism was statistically significant in diabetic obese individuals (*p* = 0.045), with the T allele being higher in the control group compared to the diabetic obese group. This suggests that the T allele may have a protective effect against diabetic obesity. When comparing the diabetic obese group with the control group, no significant differences were observed in the genotype distributions of the rs369143448 polymorphism (*p* > 0.05), but the G allele was found to be higher in the control group. This suggests that the G allele may have a protective effect against diabetic obesity. In the rs750556128 SNP, the A allele was significantly higher in the diabetic obese group compared to the control group, and carrying the A allele was shown to increase the risk of diabetes by 1.3 times in obese individuals.

This study evaluated the effects of certain SNPs on various biochemical parameters in both diabetic obese and non-diabetic obese individuals. The findings reveal that genotype and allele variations have significant effects on different biochemical markers. Specifically, the TT genotype (rs759369504) showed significant effects on different biochemical parameters in both groups, being associated with high LDH levels in diabetic obese individuals and with high ALT levels in non-diabetic obese individuals. In non-diabetic obese individuals, since ALT levels are related to liver function and this genotype may affect liver health. In diabetic obese individuals, since LDH is an indicator of tissue damage, it is possible that this genotype may be associated with tissue damage or cell breakdown in diabetic obese individuals.

The CC genotype (rs369143448 and rs781673405) was associated with creatinine and ALT levels in non-diabetic obese individuals but showed different effects in diabetic obese individuals; no significant difference was found for the rs369143448 polymorphism, while the rs781673405 polymorphism was associated with high glucose, LDH, and HbA1c levels.

rs369143448 examined in the non-diabetic obese group, individuals with the CC genotype were found to have higher and statistically significant creatinine and ALT levels compared to those carrying the G allele. This suggests that individuals with the CC genotype may have impaired kidney function and that their liver health may be negatively affected. In the analysis of rs781673405 in the diabetic obese group, the elevated LDH level in individuals with the CC genotype suggest that this genotype may be associated with the progression of diabetes and tissue damage. In the analysis of rs781673405 in the non-diabetic obese group, individuals with the CC genotype were found to have higher and statistically significant ALT levels compared to those carrying the G allele. This suggests that individuals with the CC genotype may experience impaired kidney and liver function.

The T allele (rs12443788045) was associated with high ALT and AST levels in diabetic obese individuals. These findings suggest that the T allele may have negative effects on liver function. In addition, it showed significant differences in creatinine levels in non-diabetic obese individuals.

Some studies explained that diabetes and obesity have a risk factors for elevated ALT activity in individuals with no underlying causes of liver disease. LDH test is used as a general indicator of cell damage in the body because it helps convert glucose into usable energy for our cells and is found in many tissues. These are some of the criteria we used as a basis for selecting the biochemical parameters in our study.

The A allele (rs12443788045 and rs767450259) showed significant effects on the lipid profile and kidney function in both groups. The AA genotype and A allele (rs750556128) were associated with kidney function and lipid profile in diabetic obese individuals and showed significant effects on total cholesterol and AST levels in non-diabetic obese individuals.

Overall, these findings highlight the complex effects of genetic variations on biochemical parameters, showing that not all polymorphisms affect all parameters in the same way. The lack of a significant effect of some polymorphisms on certain biochemical parameters underscores the complexity of genotype-phenotype relationships and suggests that each polymorphism may not uniformly impact all parameters. This study shows that more research is needed by keeping the sample larger in order to better understand the relationship between genetic variations and biochemical parameters in the in non-diabetic obese individuals and to develop personalized treatment approaches. These variants have an important place in prognostic evaluation for future research and clinical applications, but in order to personalize the treatment, circulating TRAIL levels as prognostic biomarkers of the onset and progression of diabetes should be evaluated together with these results and the therapeutic potential of TRAIL should be investigated.

## Conclusion

Based on the data from our study, the genotype distribution of the TRAIL rs767450259 polymorphism was found to be statistically significant (*p* = 0.045) in diabetic obese individuals, with the T allele being more prevalent in the control group. This suggests that the T allele may have a protective role against diabetic obesity. For the rs369143448 polymorphism, the G allele was found to be higher in the control group compared to diabetic obese individuals, indicating that the G allele may also have a protective role against diabetic obesity. In the rs750556128 single nucleotide polymorphism, the A allele was found to be significantly higher in the diabetic obese group compared to the control group. Our study data suggest that carrying the A allele may increase the risk of diabetes in obese individuals by approximately 1.3 times. We believe that our study will make significant contributions to the literature and pave the way for future research in this field, as it is the first to investigate and collectively evaluate the polymorphisms rs781673405, rs1244378045, rs767450259, rs750556128, rs369143448, rs143353036, and rs759369504 of the TRAIL gene.

### Data sharing and data accessibility

All data and materials are available if requested.

## Supplementary information

Supplementary

## Data Availability

No datasets were generated or analysed during the current study.
