# Supplementary material for: Evaluation of rs781673405, rs1244378045, rs767450259, rs750556128, rs369143448, rs143353036, and rs759369504 mutations in terms of polymorphism in diabetic obese and non-diabetic obese individuals
Source: Endocrine. 2025 Feb 22;88(2):467–81. doi: 10.1007/s12020-025-04184-0 (PMC12069124; doi:10.1007/s12020-025-04184-0)
Supplement: Supplementary file 1 — Supplementary [file 12020_2025_4184_MOESM1_ESM.docx]

**Table. Comparison of genotype and allele distributions of rs781673405, rs1244378045, rs767450259, rs750556128, rs369143448, rs143353036 and rs759369504 polymorphisms in the TRAIL gene in diabetic obese subjects**

| rs | Genotype and Allel | Control | Diabetic Obese | P | CI 95% |
| --- | --- | --- | --- | --- | --- |
| rs759369504 | GG | 23 (44.3%) | 22 (37.9%) | 0,796 |  |
|  | GT | 14 (26.9%) | 17 (29.3%) |  |  |
|  | TT | 15 (28.8%) | 19 (32.8%) |  |  |
|  | G Allel | 60 (52.6%) | 61 (52.5%) | 0,658 | 0.369-1.876 |
|  | T Allel | 44 (47.4%) | 55 (47.4%) | 0,502 | 0.606-2.781 |
| rs369143448 | GG | 26 (50%) | 30 (51.7%) | 0,06 |  |
|  | GA | 21 (40.4%) | 14 (24.1%) |  |  |
|  | AA | 5 (9.6%) | 14 (24.2%) |  |  |
|  | G Allel | 73 (70.2%) | 74 (63.8%) | **0,044** | 0.111-1.005 |
|  | A Allel | 31 (29.8%) | 42 (36.2%) | 0,857 | 0.441-1.974 |
| rs781673405 | GG | 15 (28.8%) | 19 (32.8%) | 0,731 |  |
|  | GC | 16 (30.8%) | 14 (24.1%) |  |  |
|  | CC | 21 (40.4%) | 25 (43.1%) |  |  |
|  | G Allel | 46 (44.2%) | 52 (47.4%) | 0,773 | 0.418-1.911 |
|  | C Allel | 58 (55.8%) | 64 (52.6%) | 0,658 | 0.369-1.876 |
| rs767450259 | TT | 23 (44.2%) | 22 (38%) | **0,045** |  |
|  | TA | 20 (38.5%) | 14 (24.%) |  |  |
|  | AA | 9 (17.3%) | 22 (38%) |  |  |
|  | T Allel | 66 (63.5%) | 58 (50%) | **0,016** | 1.332 (0.140-0.837) |
|  | A Allel | 38 (36.5%) | 58 (50%) | 0,502 | 0.606-2.781 |
| rs750556128 | AA | 22 (42.3%) | 35 (60.3%) | 0,131 |  |
|  | AG | 16 (30.8%) | 10 (17.2%) |  |  |
|  | GG | 14 (26.9%) | 13 (22.4%) |  |  |
|  | A Allel | 60 (57.7%) | 80 (69%) | **0,01** | 1.37 (0.131-0.776) |
|  | G Allel | 44 (42.3%) | 36 (31%) | 0,391 | 0.64-1.19 |
| rs1244378045 | TT | 28 (53.8%) | 32 (55.2%) | 0,506 |  |
|  | TA | 15 (28.8%) | 12 (20.7%) |  |  |
|  | AA | 9 (17.3%) | 14 (24.1%) |  |  |
|  | T Allel | 71 (68.3%) | 76 (65.5%) | 0,379 | 0.258-1.679 |
|  | A Allel | 33 (31.7%) | 40 (34.5%) | 0,889 | 0.447-2.010 |
| rs143353036 | AA | 27 (51.9%) | 32 (55.2%) | 0,584 |  |
|  | AG | 14 (26.9%) | 11 (19%) |  |  |
|  | GG | 11 (21.2%) | 15 (25.8%) |  |  |
|  | A Allel | 68 (65.4%) | 75 (64.6%) | 0,562 | 0.317-1.869 |
|  | G Allel | 36 (34.6%) | 41 (35.4%) | 0,733 | 0.414-1.859 |
